# Melatonin as a rational alternative in the conservative treatment of resistant hypertension

**DOI:** 10.1038/s41440-019-0318-3

**Published:** 2019-09-13

**Authors:** Fedor Simko, Russel J. Reiter, Ludovit Paulis

**Affiliations:** 1 Institute of Pathophysiology, Faculty of Medicine, Comenius University, 81108 Bratislava, Slovakia; 2 3rd Department of Internal Medicine, Faculty of Medicine, Comenius University, 83305 Bratislava, Slovakia; 3 Institute of Experimental Endocrinology, Biomedical Research Center, Slovak Academy of Sciences, 84505 Bratislava, Slovakia; 4 Department of Cellular and Structural Biology, UT Health, San Antonio, TX USA; 5 Institute of Normal and Pathological Physiology, Center of Experimental Medicine, Slovak Academy of Sciences, Bratislava, Slovakia

Hypertension is the most frequent cardiovascular pathology, afflicting approximately one-third of the population, and its prevalence is rapidly increasing with higher age. The term essential or idiopathic hypertension reflects its unknown etiology. However, decades of research have disclosed the apparent mechanisms leading to essential hypertension and involve endothelial dysfunction, increased oxidative load, neurohumoral imbalance, kidney alterations, or genetic predisposition. What remains truly idiopathic is the level of participation of one or several potential etiologic factors in hypertensive individuals during a particular period of hypertension development. In other words, what renders hypertension as essential is our inability to discern these factors in a particular patient. This is subsequently associated with uncertainty in the choice and dosing of antihypertensive drugs, resulting in poor hypertension control worldwide.

Treatment-resistant hypertension is when blood pressure (BP) remains above the goal despite the use of three antihypertensive drugs of different classes at optimal doses or when target values of BP are achieved only by four or more antihypertensive medications [1]. Resistant hypertension carries greater adverse cardiovascular risk than that of controlled nonresistant hypertension. Based on large clinical trials, the prevalence of resistant hypertension varies from 10 to 30% and increases with the age of the patients and the duration of hypertension [1]. Although a spectrum of antihypertensive agents is accessible, their inadequate effectiveness, side effects and nonadherence stimulated the introduction of invasive approaches, such as selective renal sympathetic denervation. However, interventional therapy requires a special team and devices, is expensive and results in the loss of counterregulatory adaptive mechanisms against potential hypovolemia; as a result, only a minority of patients with treatment-resistant hypertension are satisfactory candidates for this therapy [1]. Thus, the search after novel conservative treatment options is unremitting.

N-acetyl-5-methoxytryptamine (melatonin) was discovered as the secretory product of the vertebrate pineal gland. It is present in unicellular organisms, fungi, plants, and all animals [2, 3]. This extensive distribution suggests the hypothetic view that primitive bacteria, which evolved melatonin as an antioxidant defense mechanism, were engulfed by early prokaryotes and developed into mitochondria or chloroplasts. In the phylogenetic development from unicellular to multicellular organisms, melatonin spread to all organisms with subsequent modification of biosynthetic pathways, sites of generation, and functional implications [3]. Melatonin is a pleiotropic molecule that exerts a variety of receptor-dependent and receptor-independent biological effects. The principle receptor-dependent melatonin action is to coordinate the circadian rhythms of various physiological functions. The regulation and coordination of biological rhythms is based on the mutual interactions between the master clock (the suprachiasmatic nucleus (SCN)) in the hypothalamus and several areas in the central nervous system, as well as in peripheral tissues. The information related to light wavelength and intensity is sensed by the retina, transferred to the SCN, and transmitted to the pineal gland, where it controls melatonin production in terms of elevated melatonin secretion during darkness [4].

The broad antioxidant effects of melatonin are well recognized. This indoleamine limits oxidative stress both extracellularly and intracellularly by a variety of mechanisms, such as direct radical scavenging, stimulating the activity and expression of antioxidative enzymes, supporting glutathione synthesis and recycling, protecting other antioxidants and downregulating pro-oxidant enzymes. Melatonin also improves mitochondrial electron transport and energetic gain and modifies inflammation and apoptosis. These subcellular protective actions of melatonin result in a number of potentially beneficial actions in various systems, including a BP-reducing effect [4]. Whether melatonin can exert protection in the treatment of resistant hypertension has not been investigated.

The two-kidney one-clip model of hypertension, introduced by Goldblatt almost a century ago, is induced by the narrowing of one renal artery inducing unilateral renal ischemia with the activation of the renin–angiotensin system (RAS). Recent experiments disclosed the complex nature of this model, represented by renal hypoperfusion, oxidative stress stimulation, and sympathetic nervous system (SNS) and RAS activation. The crosstalk between the brain and kidney is impressive [5]. Kidney efferent sympathetic fibers stimulate renal artery vasoconstriction, renin release from juxtaglomerular cells and sodium and water reabsorption by the tubular system. Ischemic kidney (IK) afferent nerves modulate neurotransmission in brain areas involved in cardiovascular control. The hypothalamic paraventricular nucleus (PVN) and brainstem rostral ventrolateral medulla (RVLM) are supposedly influenced by tissue and/or circulating angiotensin II (Ang II), resulting in a sequence of events in transcriptional factor synthesis that alter the passage of impulses in baroreflex sympathetic neurons [6]. Indeed, angiotensin type 1 receptors (AT1R) and oxidative stress were shown to be increased in RVLM along with baroreflex disturbance and BP enhancement [5]. Moreover, the renal density of AT1R and urinary angiotensinogen was increased in the 2K1C model [5]. Although 2K1C is a model of secondary hypertension, due to its complex pathophysiology, this model bears considerable resemblance to resistant hypertension in clinical conditions.

The attractive and stimulating recent works of Nishi et al. [5, 7]. showed that alterations in the 2K1C model were partly reversed either by unilateral sympathetic denervation or by melatonin treatment. These authors demonstrated that the unilateral renal denervation of IK reduced the mean arterial BP and renal and splanchnic activity of SNS (rSNS, sSNS) in the contralateral kidney, with a reduction in AT1R and oxidative stress in IK and kidney protection in terms of preserving structure and reducing proteinuria. Moreover, oxidative stress and the AT1 and AT2 receptor numbers were reduced in the PVN and RVLM in the 2K1C model [5]. As such, these results support the idea that 2K1C hypertension is renovascular in its etiology but neurogenic in its mechanisms. Subsequently, the hypothesis of central modulation/treatment of this type of hypertension emerges. Indeed, gavage treatment by melatonin (30 mg/kg/day) for 15 days after the 5-week clipping of the left renal artery, prominently reduced mean arterial pressure (by 45 mmHg), attenuated sympathoexcitation to the IK, and normalized cardiac baroreflex gain and rSNS answer. Renal protection was reflected by the reduction in oxidative stress in IK and the lowering of proteinuria [7]. Yet, the study still leaves a gap in the knowledge. The principal unanswered question is where the primary action of melatonin took place. The central modulation of sympathetic outflow remains the most plausible option. However, a direct renoprotective effect of melatonin with subsequent reduced afferent stimulation from the kidney could also be postulated. Moreover, to document the potential interference of melatonin with the RAS, the Ang II levels and AT1R number in the brain and kidney should be investigated. It might also be valuable to determine the serum aldosterone level or AT1R number in the renal cortex, since aldosterone seems to play an important role in resistant hypertension. Furthermore, serum or tissue melatonin concentrations would indicate whether exogenous melatonin compensated for the potential melatonin deficit. These areas remain a challenge for future experiments.

**Fig. 1:**
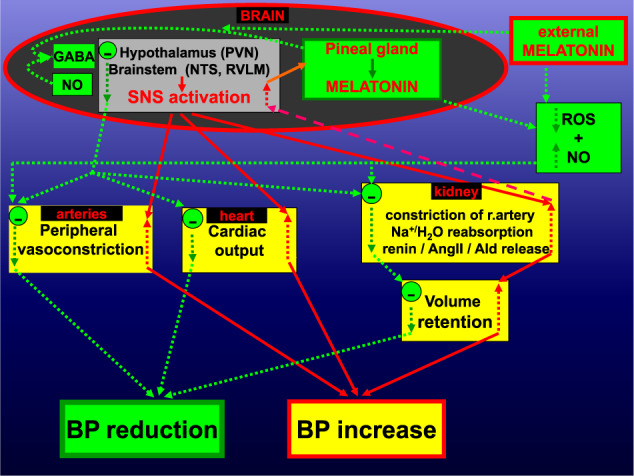
The paraventricular nucleus (PVN) of the hypothalamus and rostral ventrolateral medulla (RVLM) and nucleus of the solitary tract (NTS) of the brainstem participate in the activity of the sympathetic nervous system (SNS). The sympathetic impulses from the intermediolateral cell column increase the peripheral vascular tone and cardiac output and modify kidney function in terms of volume retention, all of which result in an enhancement of blood pressure (BP). Kidney alterations can stimulate SNS. Endogenous or exogenous melatonin may attack two principal factors of resistant hypertension: SNS and circulating volume overload. Endogenous melatonin, which is stimulated by sympathetic flow via superior cervical ganglia, exerts a negative feedback effect on SNS via γ-aminobutyric acid (GABA) inhibitory signaling on PVN, reducing the sympathetic outflow to peripheral arteries, heart and kidneys. Exogenous melatonin may carry analogical action in the SNS and can also increase the bioavailability of nitric oxide (NO) with peripheral and renal artery vasodilatation, resulting in BP reduction

In terms of the recent experimental findings and depending on the answers to the open questions, the following mechanisms could emerge (Fig. 1):

- First, endogenous melatonin production in the pineal gland is bound with the SNS. The axons of neurons in the PVN project to the preganglionic sympathetic neurons of the cervical intermediolateral cell column controlling vascular tone and arterial BP. The sympathetic impulses from intermediolateral column cells simultaneously project to the superior cervical ganglia to eventually stimulate melatonin production by the pineal gland via beta and alpha-1 adrenoceptor activation. Melatonin may have a negative feedback effect on the sympathetic system by GABA-ergic inhibitory signaling on PVN via SCN, the action of which may be potentiated by nitric oxide. Thus, endogenous melatonin may represent a counterregulatory mechanism against excessive sympathetic stimulation [8, 9]. Moreover, Melo et al. [10]. revealed in 2K1C rats that the AT1R inhibitor losartan or the activation of GABAergic receptor in the commissural nucleus (cNTS), a part of the NTS, reduced ROS, inflammation and microglia in the cNTS along with BP reduction. Since exogenous melatonin readily crosses the brain barrier [11], it may inhibit the NTS by augmenting GABAergic neurotransmission and by its extraordinary antioxidative actions to attenuate also other sympathetic loci regulating BP. Given that alpha- and beta- blockers are seldomly used because of side effects or metabolism disruptions, melatonin's sympatholytic action could be of principal value.
- Second, melatonin exerts protection in various models of kidney damage, including hypertensive and diabetic nephropathy, renal damage induced by toxic substances or invasive interventions [2]. RAS seems to play an important role in chronic kidney disturbances. The impaired melatonin secretion is associated with increased nighttime activation of local-renal RAS and renal damage in patients with chronic kidney disease. Moreover, the administration of melatonin improved structure and function in animal models of renal damage, which was associated with attenuation of intrarenal RAS activation and oxidative stress [12]. Preserving kidney function could attenuate the volume overload and hyperdynamic mechanisms of resistant hypertension development.

Third, melatonin, via its antioxidant and antiproliferative effects [13], may improve the endothelium-dependent and smooth musculature-dependent dilation of arterioles, resulting in a reduction of peripheral vascular resistance.

Since the BP threshold for starting antihypertensive therapy was reduced to ≥130/80 mmHg in the AHA guidelines, the prevalence of patients with treatment-resistant hypertension is likely to increase. Thus, finding a novel therapeutic option is a challenging issue. Commonly used therapeutic regimes involve volume reducing agents, inhibitors of the formation or effects of Ang II, and calcium channel blockers [1]. While the treatment-resistant hypertension often contains a central neurogenic pathomechanical component, the above postulated mechanisms of action of melatonin could find their application in its treatment. Based on the 2K1C model and other models of hypertension, melatonin triggers sympatholytic and bradycardic effects [9] and, due to its apparent antiproliferative action [13], this hormone even offers nephro- and cardioprotection beyond BP reduction. Therefore, melatonin becomes an essential candidate to be investigated for the treatment of resistant hypertension.
